# Whole body analysis of functional communities and topological features of gait with different speeds in Parkinson’s disease

**DOI:** 10.1007/s00415-026-13920-z

**Published:** 2026-06-09

**Authors:** Karolina Saegner, Pedro Conceição, Robbin Romijnders, Julius Welzel, Nicolas Vuillerme, Clint Hansen, Elke Warmerdam, Walter Maetzler

**Affiliations:** 1 Department of Neurology, University Hospital Schleswig-Holstein, Campus Kiel and Kiel University, Arnold-Heller Str. 3, 24105 Kiel, Germany; 2 Institute of Computer Science, Dependable Systems, Kiel University, Kiel, Germany; 3 Department of Psychology, Carl von Ossietzky Universität Oldenburg, Oldenburg, Germany; 4 Univ. Grenoble Alpes, CNRS, Grenoble INP, LIG Sangria, Grenoble, France; 5 Institut Universitaire de France, Paris, France; 6 Biomedical Signals and Systems, Faculty of Electrical Engineering, Mathematics and Computer Science, University of Twente, Enschede, The Netherlands

**Keywords:** Gait analysis, Graph, Kinectome, Network

## Abstract

**Background:**

During human gait, different body segments work coherently to achieve forward propulsion in a coordinated manner. It is known that Parkinson’s disease (PD) disturbs this coordination, however, which body segments contribute most, especially with changing walking speed, has not been evaluated.

**Objectives:**

To investigate in people with PD (PwPD) and healthy controls how individual body segments contribute to altered gait during different walking speeds.

**Methods:**

Twenty-nine PwPD and 29 controls walked forward along a straight walking path at three walking speeds. For each speed and movement direction, kinectomes were built by computing pairwise correlations between body segment accelerations. Network graphs were generated with anatomical segments as nodes and their co-accelerations as edges. Graph-theoretic analysis examined community organisation, modularity metrics, and network topology. Nodal strength, defined as coherence between a body segment and all other body segments, was extracted.

**Results:**

Compared to controls, PwPD showed coherence deficits during walking, particularly at preferred speed and in the anteroposterior direction, and these deficits primarily affected the core body segments. Controls demonstrated speed-dependent modulation of coherence that was absent in PwPD, particularly in the anteroposterior direction within core body segments. Moreover, PwPD showed speed-dependent modulation of coherence primarily in lower limb segments across the mediolateral and vertical directions.

**Conclusion:**

In PwPD, gait coordination deficits localise primarily to the core body segments and are accompanied by a reduced ability to modulate whole-body coordination with walking speed, suggesting that trunk-focused and progressive speed-variable gait training may be a particularly effective rehabilitation strategy. The graph-theoretical framework further enables individualised identification of segmental coordination deficits for tailored treatment planning.

**Trial registration:** The study is registered in the German Clinical Trials Register (DRKS00022998, registered on 04 Sep 2020).

**Supplementary Information:**

The online version contains supplementary material available at 10.1007/s00415-026-13920-z.

## Introduction

Parkinson’s disease (PD) is one of the most common neurodegenerative diseases worldwide [1]. It manifests with a variety of non-motor and motor symptoms, the latter including bradykinesia, tremor, rigidity, and unstable posture [2]. The clinical severity and progression of PD are commonly classified using the Hoehn and Yahr (H&Y) scale, which ranges from stage 1 (unilateral involvement with minimal functional impairment) to stage 5 (severe disability) [3]. Importantly, motor symptoms in PD typically appear asymmetrically, with unilateral onset reported in over 65% of individuals [4]. This segmental asymmetry is particularly characteristic of early-stage disease (H&Y stage 1–2), with research showing that asymmetry in gait parameters can be detected already at these early stages, preceding the broader gait impairments that emerge at more advanced stages [5, 6]. Furthermore, people with PD (PwPD) experience difficulties with gait, such as decreased stride length and velocity [7], impaired foot placement [8], reduced gait speed [9, 10] and increased cadence and double support time [9], imposing difficulties on daily life activities [11].

Walking is defined as a continuous forward progression of the centre of mass in a desired direction. Propulsion and support are provided by the two lower limbs, which move in a reciprocal cycle of lifting and landing, with one foot staying in contact with the ground at all times [12]. In addition, the two upper limbs swing contralaterally, such that each upper limb moves forward together with the opposite lower limb [13]. Therefore, different musculoskeletal segments work simultaneously in a coordinated manner, and an accurate overall characterisation of gait requires a view on the entire human body as a complex and integrated system with many interacting subsystems at varying levels of complexity [14]. For this purpose, network theory can be used, allowing one to investigate complex systems, consisting of many interconnected elements, and their interactions with each other [15, 16]. This provides insights not only into the overall behaviour of the whole system, but also into the particular roles each element can have within the network [17].

Troisi Lopez et al. [18], using kinematic data of different body segments, introduced a *kinectome* framework to characterise the kinematics of human gait. This allowed large-scale descriptions of gait with regards to interactions between different body segments, as well as examination of single body segment involvement in the movement. Using modularity analysis [18], these authors found an asymmetrical functional organisation of the body segments in PwPD, as compared to controls. With the topological analysis, the authors could show that PwPD have less rigidity in the head and trunk, as well as improved coupling between upper and lower limbs after intake of levodopa, compared to the medication off state [19]. Importantly, these results also showed that this type of analysis allows assessment of the role of each body segment with respect to the entire body and all the other body segments [20].

However, to the best of our knowledge, in PwPD, the changes in the level of coupling of body segments during walking at different speeds have not been evaluated. Gait impairment in PwPD is well recognized as a multifactorial phenomenon, arising from disrupted neural circuitry, reduced muscle strength and forward propulsion, as well as psychological factors, such as fear of falling [21]. In our previous work, we have shown that PwPD cannot adapt their lower limb angles like controls to changing walking speed requirements [22], reflecting one of many contributing biomechanical manifestations of this broader dysfunction. Moreover, we could show that the inter-segmental coordination of PwPD is affected on multiple levels, ranging from coupling between a few segments to whole-body coordination [23]. Investigating the relative importance of each body segment at different walking speeds, and comparing this with that of healthy adults, could improve our understanding of the underlying mechanisms of gait dysfunction in PwPD. It could also help us to identify the body segments that contribute most to walking impairments at different speeds and inform novel training strategies. For example, findings from the present analysis could inform speed-based gait training protocols, in which PwPD practice walking across a range of speeds to systematically challenge the speed-dependent modulation of intersegmental coordination—an approach that conventional cadence- or stride-length-focused therapies do not explicitly target. Therefore, our aim was to examine how the relative contribution of individual body segments to walking changes with varying walking speeds in PwPD compared to controls. We hypothesized that PwPD shows a reduced ability to modulate the relative contribution of individual body segments to walking coordination with changing walking speed.

## Methods

### Ethics approval and consent to participate

The study was conducted in accordance with the principles of the Declaration of Helsinki and was approved by the ethics committee of the Medical Faculty of Kiel University (D438/18). The study is registered in the German Clinical Trials Register (DRKS00022998, registered on 04 Sep 2020). Prior to the study, all participants have read and signed informed consent.

### Participants, in- and exclusion criteria

Study participants needed to be at least 18 years old and able to walk without assistance. Exclusion criteria included Montreal Cognitive Assessment scores below 15 or other movement disorders than PD affecting mobility (assessed by a movement disorder specialist, WM). Full selection criteria are detailed in [24]. The analysis examined 58 individuals, 29 PwPD (11 females) and 29 age-matched controls (14 females). PwPD were assessed during self-identified optimal medication state (30–120 min post levodopa intake). Controls had no mobility-affecting conditions (verified by WM). In PwPD, 11 participants had the most affected upper and lower extremities on the same side of the body, 2 had cross lateralisation. Three had a most affected upper limb, and 2 had a most affected lower limb. Details are shown in Table 1.

**Table 1:** Demographic and clinical parameters of the participating groups

Group	Controls	PD	*p*-value
Total [*N*] (males/females)	29 (15/14)	29 (18/11)	0.426
Age [years]	67 ± 12	67 ± 10	0.479
Height [cm]	176 ± 10	174 ± 9	0.669
Weight [kg]	79 ± 16	82 ± 18	0.477
BMI [kg/m^2^]	26 ± 5	27 ± 5	0.341
Disease duration [years]	–	9 ± 6	–
Hoehn & Yahr (1–5)	–	3 ± 1	–
Medication dose [LEDD]	–	713 ± 301	–
MDS-UPDRS III (0–132)	4 ± 4	29 ± 21	< 0.001
Gait speed (preferred) [m/s]	1.11 ± 0.26	0.95 ± 0.20	< 0.05
Gait speed (fast) [m/s]	1.54 ± 0.26	1.29 ± 0.29	< 0.01
Gait speed (slow) [m/s]	0.63 ± 0.19	0.64 ± 0.21	0.738

The values are displayed as mean ± standard deviation. The *p*-value column indicates the differences between the groups

*BMI* body mass index, *LEDD* levodopa equivalent daily dose, *MDS-UPDRS III* Motor part of the Movement Disorder Society Unified Parkinson’s Disease Rating Scale, *PD* Parkinson’s disease

### Most and least affected body sides

Most and least affected extremities were identified using MDS-UPDRS III motor scores [25], combining relevant items for upper (3.3–3.6) and lower (3.3, 3.7–3.8) extremities. In PwPD, lateralisation required ≥ 1 point difference [26]. In cases where bilateral scores were equal, and in controls, the non-dominant side was designated as most affected [27]. Detailed methodology is available in [23].

### Motor assessment, equipment and data acquisition

Participants walked forwards a 5-m walkway at three speeds: preferred (“Please walk at your normal walking speed”), slow (“Please walk half of your normal walking speed”), and fast (“Please walk as fast as possible, without running, falling or feeling unsafe”). Trials started 2 m before and ended 2 m after the walkway to ensure steady-state walking during the gait assessment phase [10, 28].

Walking was recorded using a twelve-camera motion capture system operating at 200 Hz (Qualisys AB, Göteborg, Sweden) with 47 reflective markers (Suppl. Figure 1a). Complete procedures are available in [24].

### Marker data pre-processing

**Fig. 1:**
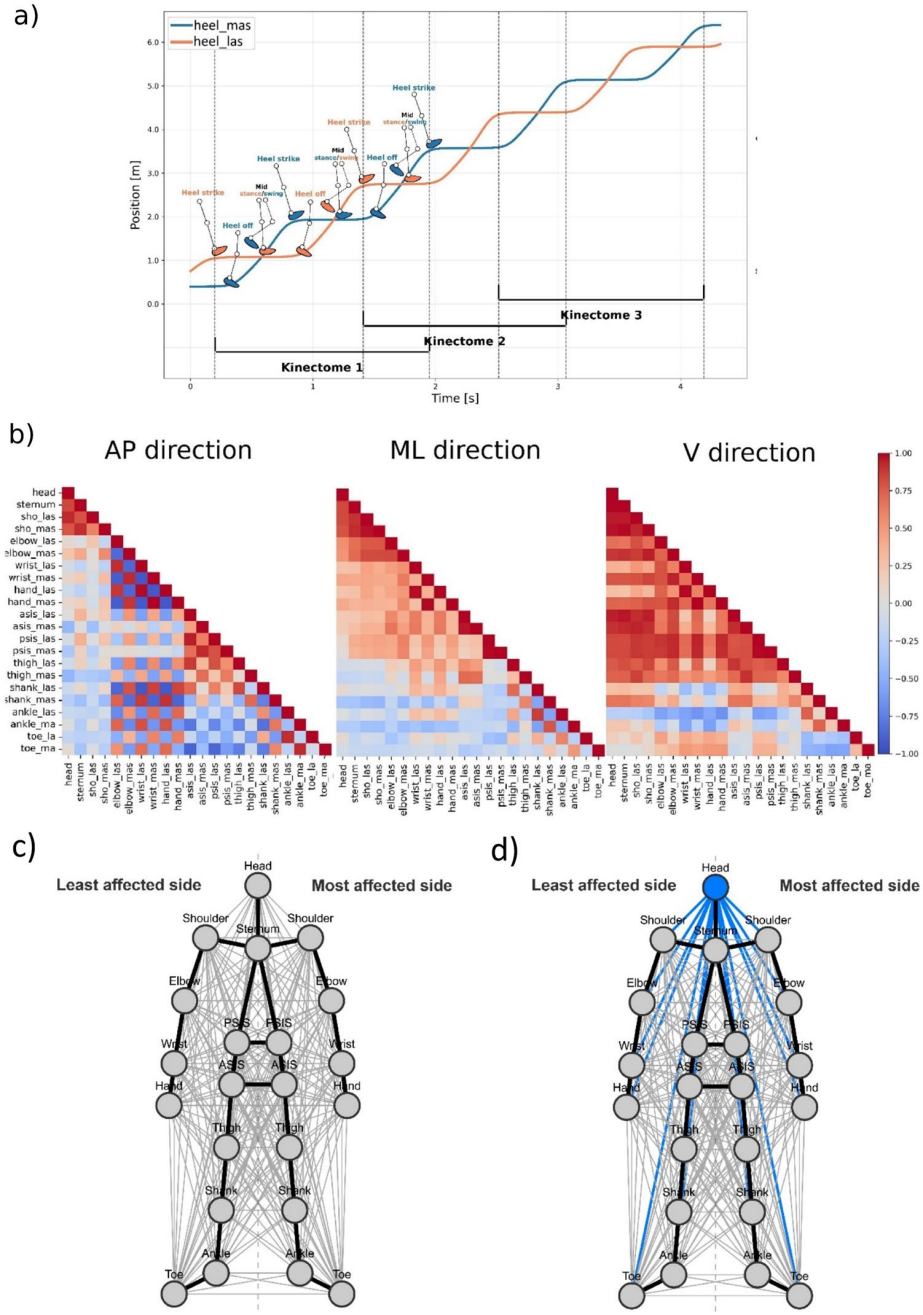
Scheme of the kinectome analysis. **a** Position change in the anteroposterior direction of the most and least-affected heels; data cut into one full left and one full right gait cycle, respectively = one gait acquisition kinectome; **b** Example kinectomes in anteroposterior, mediolateral, and vertical directions; **c** Representation of the kinectome as a graph; **d** All edges of a node are highlighted. *Asis* anterior superior iliac spine, *las* least affected side, *mas* most affected side, *psis* posterior superior iliac spine

The 47 reflective markers were reduced to 22 locations representing the body segments for analysis (Suppl. Figure 1b) [23]. Source data were converted to the Brain Imaging Data Structure (BIDS) [29, 30] and pre-processed using Python (Python Software Foundation. Python Language Reference, version 3.11.2). One complete stride cycle for each leg was extracted (Fig. 1a), linearly interpolated for missing data (maximum gap 1.35 s) using Kinetics Toolkit’s fill_missing_samples function [31], and filtered (Butterworth low-pass filter, 2nd order, 6 Hz). Principal component analysis was used to align the principal movement axis (anteroposterior (AP) movement direction) with the X axis of the laboratory’s global coordinate system. Then, positional data were double-differentiated to derive accelerations.

### Kinectome analysis

Acceleration time series from each marker were extracted in AP, mediolateral (ML), and vertical (V) movement directions, and cut to contain one full left and one full right gait cycle in one gait acquisition (Fig. 1a, dashed lines) [32]. Each such segment of data, spanning one full bilateral gait cycle, was normalised to 500 points and used to compute a single kinectome via Pearson correlations between all marker pairs [18]. Each kinectome is derived from the acceleration time series of a single gait acquisition window (e.g., Kinectome 1, 2, or 3 in Fig. 1a) and captures the pairwise correlation structure across all body segments at that moment in time. This produced three 22 × 22 correlation matrices (22 body segments across 3 movement directions, Fig. 1b) for each trial. Participant kinectomes (at least three per trial and per direction) were derived by averaging multiple gait acquisition kinectomes (e.g., averaging kinectomes one, two, and three in Fig. 1a) within each trial and direction, yielding a single, participant-specific average kinectome per speed and direction that represents the coordination structure of that individual’s gait. This resulted in 9 kinectomes (AP, ML, and V, for each of the three walking speeds) per participant. Each kinectome was converted to a fully-connected, undirected graph (Fig. 1c) [18], where each node represents a body segment and each weighted edge reflects the magnitude of the acceleration correlation between two segments. Figure 1c and d, therefore, depict the graph representation of this averaged participant-specific kinectome, and are derived from—not directly annotated in—the temporal windows shown in Fig. 1a. Graph analyses were done using the NetworkX Python package (version 3.4.2) [33].

### Functional community detection

The methods to detect functional communities (FCs) typically assume that there is a natural division of the nodes within the network into smaller subgroups, whose size is determined by the network itself [16, 34]. A graph is said to have FC structure if the nodes can be grouped into sets of nodes such that each set is densely connected internally and has sparser between-group connections [15, 35, 36]. In human movement, FCs represent body segments working together in a coordinated manner to perform a motor task. In gait analysis, this approach allows to identify which body segments exhibit coordinated movement patterns, revealing the underlying coordination strategies used during walking. Given that all body segments in the human body are connected [37], there are just as many connections between the FCs, as within the FCs.

We evaluated these FC structures in the whole body for each participant with the Louvain method using networkx.louvain_communities with consensus clustering across 100 iterations to determine which body segments belong to the same FCs [18]. Allegiance matrices containing probabilities that two segments cluster together over multiple iterations were calculated for each participant’s kinectome.

### Modularity analysis

Modularity quantifies the strength of the division of a network into FCs, answering the question whether these FCs are coordinated together stronger than could be expected by chance. Values range from − 1 to 1: negative values indicate worse-than-random grouping, ~ 0 indicates random grouping, and positive values indicate real coordination, i.e., body segments within each group move together in a coordinated manner more than is expected by chance [34]. In the context of whole body gait analysis, positive modularity indicates that the observed FC structure is biomechanically meaningful. This value was calculated using NetworkX (nx.community.modularity) with varying resolution, which refers to the “zoom level” of the analysis. Low resolution favours larger FCs (gross movement patterns), while higher resolution favours smaller FCs (finer movement patterns) [16, 33]. We focused here on the whole-body analysis, i.e., calculating modularity for the FC structure of the entire (whole body) network.

The threshold (two nodes belong to the same community if their allegiance score > threshold) was set to 0.6. Communities were detected at the individual participant level, that is, each participant’s kinectome was partitioned independently, yielding a participant-specific community structure. These were then averaged between the groups to obtain the community structure for each group (separately for the three walking speeds and the three movement directions).

### Topological analysis

To assess the importance of each node within the network, a weighted degree centrality [38] was calculated by summing the absolute values of all edge weights connected to that node, which is referred to as nodal strength, as follows (1):

1 $${S}_{i}= \sum_{j=1}^{\begin{array}{c}i\ne j\\ N\end{array}}\left|{W}_{ij}\right|,$$

where $i$ and $j$ are two nodes from the network, $W$ is their connecting edge, and $N$ is the number of nodes in the whole network [19] (here – whole body). A graphical example representation can be found in Fig. 1d. This allowed a more granular evaluation of the relative importance of each body segment with respect to the whole body [19, 38] in each walking speed and movement direction.

We refer to the nodal strength as coherence (formerly called “synchronisation” [19]) between a body segment and all other body segments during gait. Higher coherence indicates stronger coordinated movement with the rest of the body; weaker coherence indicates a more decoupled movement.

### Statistics

Statistical analysis was done using SciPy [39] and JASP (JASP Team, version 0.18, University of Amsterdam, The Netherlands). Shapiro–Wilk test was used to check if the data are normally distributed. Outliers were removed using *Z*-score (removing values ± 3 SD from the group mean) for normally distributed data, or by removing values 1.5 times greater or less than the interquartile range for non-normally distributed data. The gender distribution between the groups was compared using the Chi-square test. All other variables (values from modularity and centrality analyses) were compared between the two groups within the same speed and movement direction using *t*-tests and Mann–Whitney *U* tests for normally and non-normally distributed data, respectively. To adjust for multiple comparisons, the False Discovery Rate method (*p* < 0.05) [40] was used. Comparisons between the three walking speeds were done using repeated measures ANOVA (with Bonferroni post-hoc correction) or Friedman test (Conover’s post hoc correction) for normally and non-normally distributed data, respectively. The robustness of the topological analysis was evaluated through bootstrap resampling (*n* = 1000) at 80% of the sample size. The metrics were calculated separately for each walking speed and movement direction. For easy readability, all data are presented with mean ± standard deviation (SD).

## Results

### Functional communities

#### Selection of the functional community structure

**Fig. 2:**
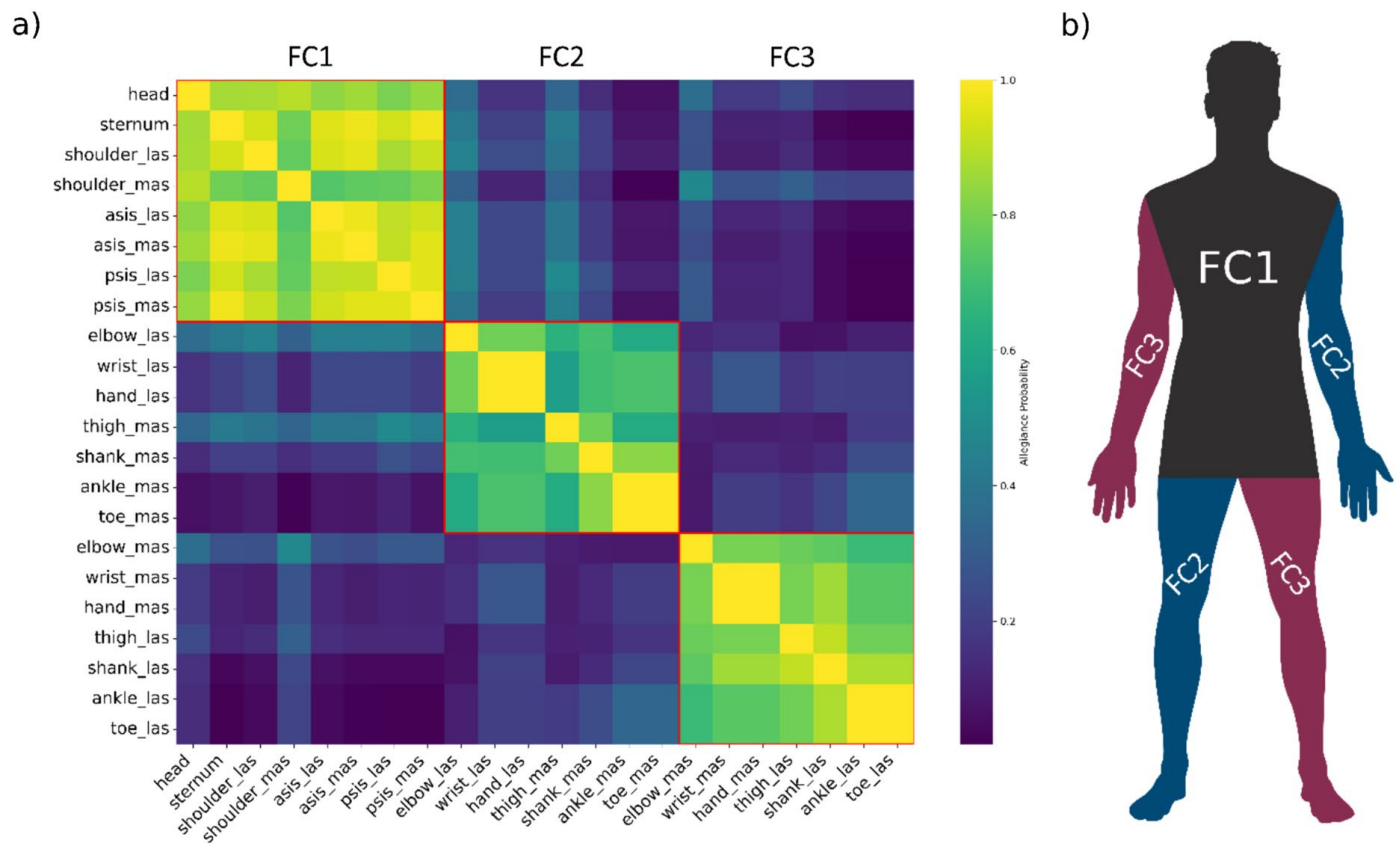
Functional communities during gait. **a** Allegiance matrix; **b** Functional communities. Functional Community 1 includes core segments, Functional Community 2 includes the least affected upper limb and most affected lower limb, and Functional Community 3 includes the most affected upper limb and the least affected lower limb. *Asis* anterior superior iliac spine, *FC* functional community, *las* least affected side, *mas* most affected side, *psis* posterior superior iliac spine

We used the data from the control group at preferred walking speed in the AP direction to define our reference framework for the FC structure (Fig. 2). One FC (further referred to as FC1) involved the core body segments, the head, sternum, shoulders, and all four pelvic segments. The second FC (further referred to as FC2) involved the least affected elbow, wrist, and hand and the most affected thigh, shank, ankle, and toe. The last FC (further referred to as FC3) involved the most affected elbow, wrist, and hand and the least affected thigh, shank, ankle, and toe. PwPD showed the same framework for the FC structure.

#### Modularity, and the influence of resolution in gait analysis

Modularity analysis did not reveal significant differences between the PwPD and controls, providing evidence that the methods used are able to detect the usually expected biomechanical aspects of human gait. The evaluation of FCs and their modularity shows that the general pattern of movement during gait is similar between PwPD and controls. Contralateral limbs swing forward in a coordinated manner while the core body segments move together during forward propulsion.

However, a strong influence of the resolution parameter for modularity values was identified. Decreasing resolution from 1.5 to 0.1 produced a systematic increase in modularity scores (from − 0.2–0 to 0.3–0.5), reflecting the algorithm’s tendency to merge smaller, weakly-connected body segment clusters into larger, more cohesive communities at lower resolution values.

### Topological analysis

#### Differences between the groups within the same walking trials

**Fig. 3:**
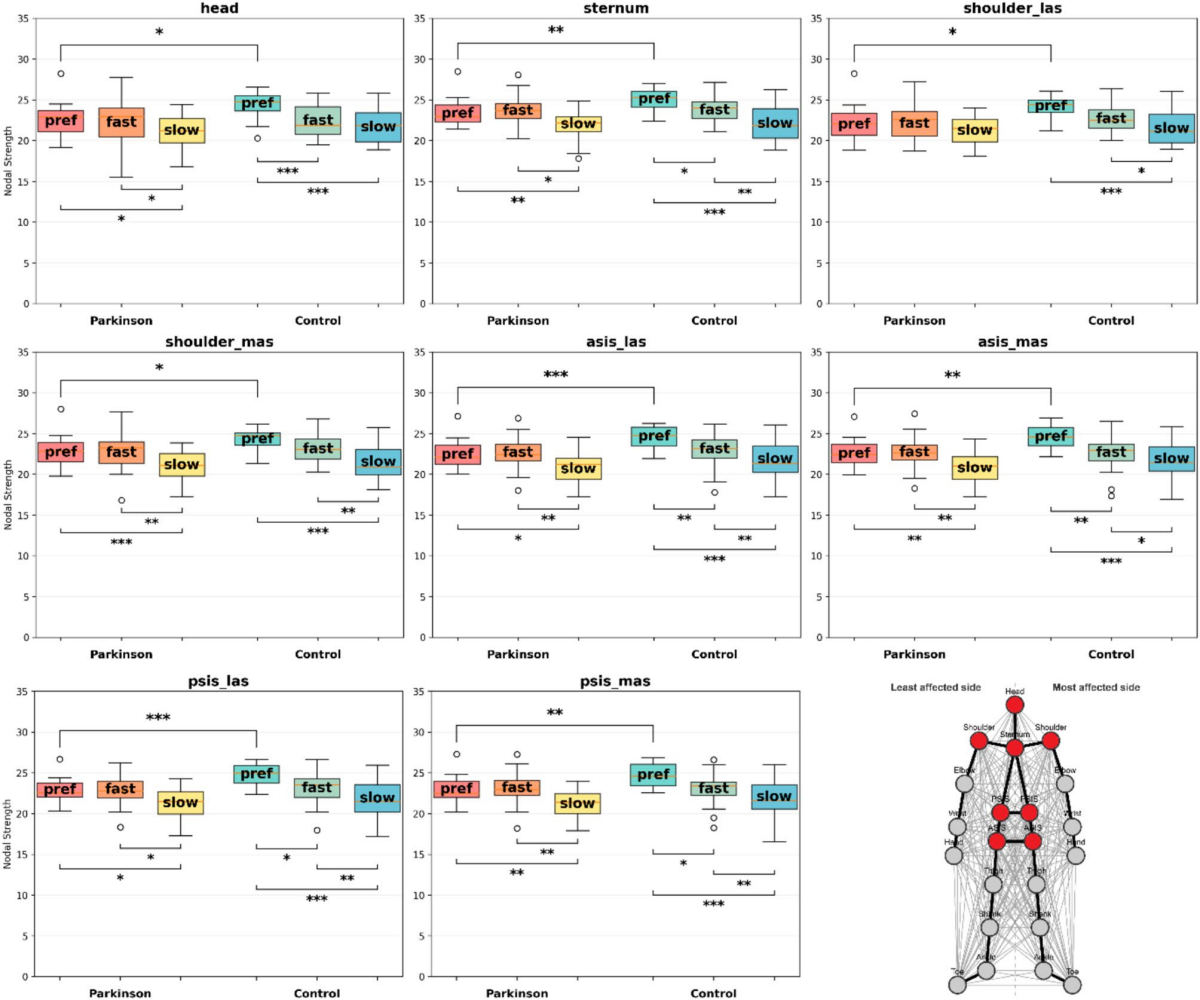
Nodal strength, reflecting the degree of coherence between a body segment and all other body segments during gait (Y axis) of the respective body segments in FC1 (core body segments) in the AP direction. The red nodes on the stick figure indicate the body segments where the differences between the groups were significant. *Asis* anterior superior iliac spine, *fast* fast walking speed, *las* least affected side, *mas* most affected side, *pref* preferred walking speed, *psis* posterior superior iliac spine, *slow* slow walking speed. *p < 0.05, **p < 0.01; ***p < 0.001

Data are presented in Fig. 3 and supplementary Figs. 2–9, of which the most relevant significant findings are summarised in the following. During preferred walking speed, in the AP direction, all body segments of FC1 (Fig. 3) and the least affected thigh and shank (FC3; Suppl. Figure 3) had less coherence in PwPD than in controls. During fast walking speed, in the V direction, the head segment had more coherence in PwPD than in controls (Suppl. Figure 7). No significant differences were observed between the groups during slow walking speed. The statistical significance of the results was maintained across bootstrap resampling iterations.

#### Associations between walking speed and body segment coherence

Data are presented in Figs. 3 and supplementary Figs. 2–9. The most relevant statistically significant results are summarised below.

***AP direction:***

*Community 1:* In both groups, all body segments except the less affected shoulder had less coherence at slow speed, compared to preferred and fast speeds. Controls showed less coherence of various segments in the fast versus preferred speed that were not observed in the PD group (Fig. 3).

*Community 2:* In both groups, lower limb segments (most affected shank, ankle, toe) had less coherence at slow speed, compared to preferred and fast speeds. In addition, the most affected ankle and toe had less coherence at preferred speed, compared to fast speed. PwPD showed less coherence in the most affected thigh at slow speed, compared to preferred speed. Controls showed less coherence in the least affected elbow at fast speed, compared to preferred and slow speeds (Suppl. Figure 2).

*Community 3:* In both groups, the least affected thigh, shank, ankle and toe had less coherence at slow speed, compared to preferred and fast speeds. In addition, in both groups, the least affected ankle and toe had less coherence at preferred speed, compared to fast speed. Controls showed less coherence in the most affected elbow at slow speed, compared to preferred speed; in the most affected wrist at slow speed, compared to preferred and fast speeds; and in the most affected hand at slow speed, compared to preferred and fast speeds, and at preferred speed, compared to fast speed (Suppl. Figure 3).

***ML direction:***

*Community 1:* Controls showed less coherence in both shoulders at fast speed, compared to preferred speed, and in the most affected shoulder at fast speed, compared to slow speed. Controls also showed less coherence in both anterior hip segments at fast speed, compared to preferred speed (Suppl. Figure 4).

*Community 2:* In both groups, the most affected ankle and toe had less coherence at slow and preferred speeds, compared to fast walking speed. PwPD showed less coherence in the most affected thigh at slow speed, compared to fast speed, and in the most affected shank at preferred and slow speeds, compared to fast speed. Controls showed less coherence in the least affected elbow at fast speed, compared to preferred speed; in the least affected wrist at slow speed, compared to fast speed; and in the least affected hand at slow speed, compared to preferred and fast speeds. In addition, controls showed less coherence in the most affected shank at slow speed, compared to fast speed; and in the most affected ankle and toe at slow speed, compared to preferred speed (Suppl. Figure 5).

*Community 3:* In both groups, the least affected ankle had less coherence at slow and preferred speeds, compared to fast speed. In addition, in both groups, the least affected toe had less coherence at slow speed, compared to preferred and fast speeds. PwPD showed less coherence in the least affected ankle at slow speed, compared to preferred speed. Controls showed less coherence in the most affected elbow at fast speed, compared to preferred speed; in the most affected wrist and hand at slow speed, compared to preferred and fast speeds; in the least affected shank at slow speed, compared to fast speed; and the least affected toe at preferred speed, compared to fast speed (Suppl. Figure 6).

***V direction:***

*Community 1:* In both groups, all the segments had less coherence at slow speed, compared to preferred and fast speeds. PwPD showed less coherence in the sternum at preferred speed, compared to fast speed (Suppl. Figure 7).

*Community 2:* In both groups, the least affected elbow, and the most affected thigh, shank, and toe had less coherence at slow speed, compared to preferred and fast speeds. In addition, in both groups, the least affected thigh and toe had less coherence at preferred speed, compared to fast speed. PwPD showed less coherence in the most affected shank at preferred speed, compared to fast speed, and in the most affected ankle at fast speed, compared to slow speed. Controls showed less coherence in the least affected hand at fast speed, compared to slow speed (Suppl. Figure 8).

*Community 3:* In both groups, the most affected elbow, and the least affected thigh, shank, and toe had less coherence at slow speed, compared to preferred and fast speeds. In addition, in both groups, the least affected thigh and toe had less coherence at preferred speed, compared to fast speed. PwPD showed less coherence in the least affected ankle at fast speed, compared to slow speed. Controls showed less coherence in the most affected wrist and hand at fast speed, compared to slow speed (Suppl. Figure 9).

Taken together, PwPD showed minimal upper limb modulation, but exhibited speed-dependent changes primarily in lower limb segments across ML and V directions. Controls demonstrated speed-dependent modulation of coherence that was absent in PwPD, particularly in the AP direction within Community 1 (core body segments). Furthermore, controls consistently showed reduced coherence of upper limb segments at slow and fast speeds compared to preferred speed across all directions. The statistical significance of the results was maintained across bootstrap resampling iterations.

## Discussion

This study describes the FC structure and the coherence of single body segments with the rest of the body during walking, in PwPD and age-matched controls. It also examines how this coherence changes at different walking speeds.

First, the FC structure found in the present analysis was the same as the FC structure in healthy controls in [18], and represents the biomechanical aspects of gait [12] during preferred, fast, and slow walking speed conditions. Second, the FC and modularity analyses confirmed that these graph-theoretical methods successfully captured gait patterns consistent with established biomechanical principles of walking. In PD, gait impairments are known to manifest as more focal, asymmetric deficits, particularly in the earlier stages of the disease, rather than as a full reorganisation of whole-body movement coordination [21]. It is, therefore, plausible that the global FC and modularity structure is preserved in our sample, while segment-specific deficits—as revealed by our node-level analyses—remain detectable. Importantly, coordination between body segments during gait occurs at multiple scales, and the choice of resolution value determines the level of coordination being investigated—from local joint couplings (high resolution factor) to global full body segmental coordination (low resolution factor). Therefore, setting the research question before choosing the resolution value and reporting it accordingly is crucial. Our data suggest that resolution factors ≤ 0.5 better capture gross inter-limb and trunk-limb coordination patterns, while values between 0.5 and 1 might reveal intra-limb segmental couplings, and values > 1 tend to over-fragment the natural biomechanical communities.

Examining these coordination patterns at a granular level through topological network analysis revealed that the majority of differences between the groups were observed during preferred walking speed (but not slow and fast) and in the AP direction in Community 1, which includes the core body segments. We assume that walking at preferred speed relies more on automatic programmes than walking slowly or fast. It is known that PD particularly affects automatic movements [41]. Walking faster than preferred speed could serve as a cue, increasing arousal and reducing automatic contribution to the movement, and thus bringing the performance of PwPD closer to those of controls. A well-known example for this is paradoxical kinesis in PwPD during dangerous situations [42, 43]. Walking slowly may also serve as a cue. Moreover, while walking slowly, PwPD might have enough time to adapt the performance of the motor system to the task requirements. This is also supported by the observed significant differences between PwPD and controls in preferred and fast walking trials in AP and V directions, respectively, which were not observable in the slow walking condition. The predominance of observed differences in the AP movement direction aligns with the biomechanical principles of human gait. The forward propulsion appears in the sagittal plane, which is where the largest displacement of body segments and velocity changes happen [44]. PD-related motor impairments, such as reduced stride length and time [7], primarily manifest in the sagittal plane, rendering differences in kinematics in the AP direction particularly discriminative between PwPD and controls.

What is, in our view, a particularly interesting result of these analyses is the dependency of body segment coherence in the AP direction on walking speed. While controls “lost”, as expected, coherence when walking slower or faster, compared to preferred walking speed, PwPD did not show this loss between preferred and fast walking speeds. This displays most probably a reduced speed-dependent adaptability of the motor system, suggesting a limit in the motor output capacity, i.e., that the motor system is working closer to its maximum performance already at preferred speed, and there is little room left to increase the motor performance when walking speed is increased. Targeted exercise programmes using, for example, progressive speed-dependent treadmill training could, therefore, be beneficial in improving gait’s adaptability in persons that show such behaviour.

PwPD, but not controls, demonstrated increased coherence of the most affected shank and thigh at fast versus preferred and slow speeds in the ML direction, with similar patterns observed in the V direction and sternum. This suggests that PwPD retains certain adaptive capacity at faster speeds, particularly in non-sagittal planes. Given that ML foot placement control is essential for gait stability and can be trained even in older adults [45], the higher ML coherence at fast speeds may reflect a compensatory strategy to maintain lateral balance. While controls showed minimal speed-related ML and V modulation in the lower limbs, PwPD appear to prioritise stabilisation in these directions at faster speeds, possibly at the expense of forward propulsion efficiency.

Furthermore, we have also observed reduced upper limb coherence differences between the speeds in PwPD. Arm swing, crucial during gait, has been shown to positively respond to dopaminergic medication, with swing amplitude and peak angular velocity increasing after the intake of medication [13]. However, we see that this improvement does not translate to scaling the upper limb movements to different gait speeds, especially in the most affected upper limb segments. Interestingly, in PwPD, the coherence differences in the lower limb between the speeds are much more pronounced. This supports previous findings about the “distribution” of hypokinesia and rigidity in PD (typically more pronounced in the upper than in the lower limbs [2]).

The methods presented in this study have promising clinical applications in movement assessment and rehabilitation planning. A practical implementation could involve establishing normative FC structures and their coordination strengths from a large control cohort. Any patient could then be evaluated using the same analytical framework and compared against these normative values, regardless of their specific condition. This comparison could be performed at multiple levels of granularity, from whole-body coordination patterns to individual FCs and specific body segments. This level of detail could provide valuable guidance for rehabilitation specialists, helping them to target interventions and identify which body segments or coordination patterns deviate most from healthy controls and, therefore, require focused therapeutic attention during treatment.

This study has strengths and limitations. Among the strengths are the evaluation of segmental coherence of the entire body during gait, done at different walking speeds. As for the limitations, the evaluation of PwPD was done only while on medication. Different body segment coherence and the influence of walking speed could be revealed in the off-medication state. Other limitations include a relatively high disease variability, the focus on relatively short walking trials without any obstacles or external disturbances, which are highly present in daily life, and a relatively high proportion of PwPD without clearly expressed lateralisation. Furthermore, a larger sample would allow a better identification of FCs in all three movement directions. Still, we feel that this pilot analysis could stimulate research in this area, bridging clinical experience with novel analysis approaches to extract more granular and meaningful data from affected persons.

## Conclusion

Gait impairment in PwPD reflects reduced coordination of the core body segments, and not of the extremities, and reflects a loss of speed-dependent modulation of whole-body coordination, with the motor system apparently operating near its capacity already at preferred speed. These findings argue for prioritising trunk stability and progressive speed-variable gait training in PD rehabilitation, domains that are also relevant to fall prevention and the preservation of mobility in daily life. The graph-theoretical analysis approach allows a deeper kinematic understanding of gait in PwPD, and how this can be investigated on multiple levels, starting from whole-body and down to individual body segments. This allows the testing and implementation of novel rehabilitation strategies for gait deficits in PD, especially on coherence deficits of various body segments during different walking speeds.

## Supplementary Information

Below is the link to the electronic supplementary material.

Supplementary file1 (DOCX 1202 KB)

## Data Availability

Part of the data analysed are available in [46]. The rest of the data contain patient information; therefore, they are available from the corresponding authors on reasonable request. The code for data analysis is available in [47] (requires Python 3.11 or higher).
