# Supplementary material for: Whole body analysis of functional communities and topological features of gait with different speeds in Parkinson’s disease
Source: J Neurol. 2026 Jun 9;273(7):381. doi: 10.1007/s00415-026-13920-z (PMC13246872; doi:10.1007/s00415-026-13920-z)
Supplement: Supplementary file 1 — Supplementary file1 (DOCX 1202 KB) [file 415_2026_13920_MOESM1_ESM.docx]

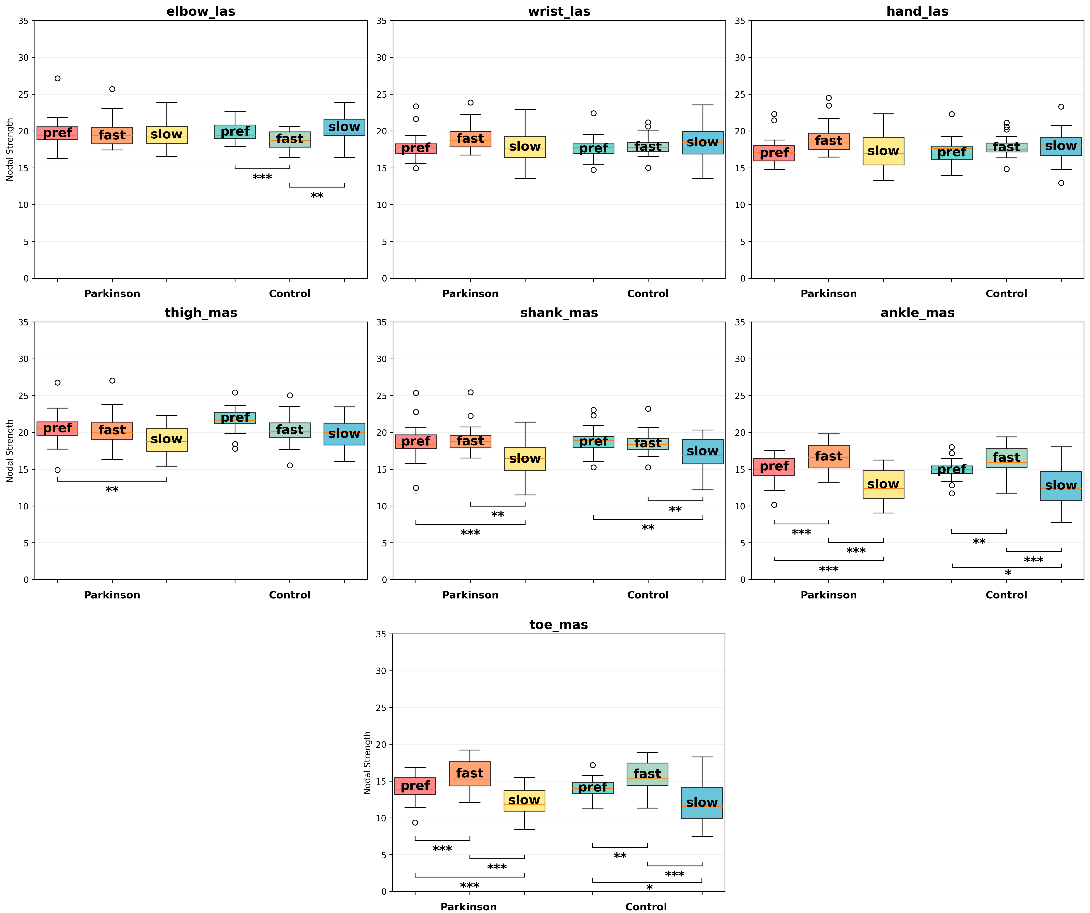

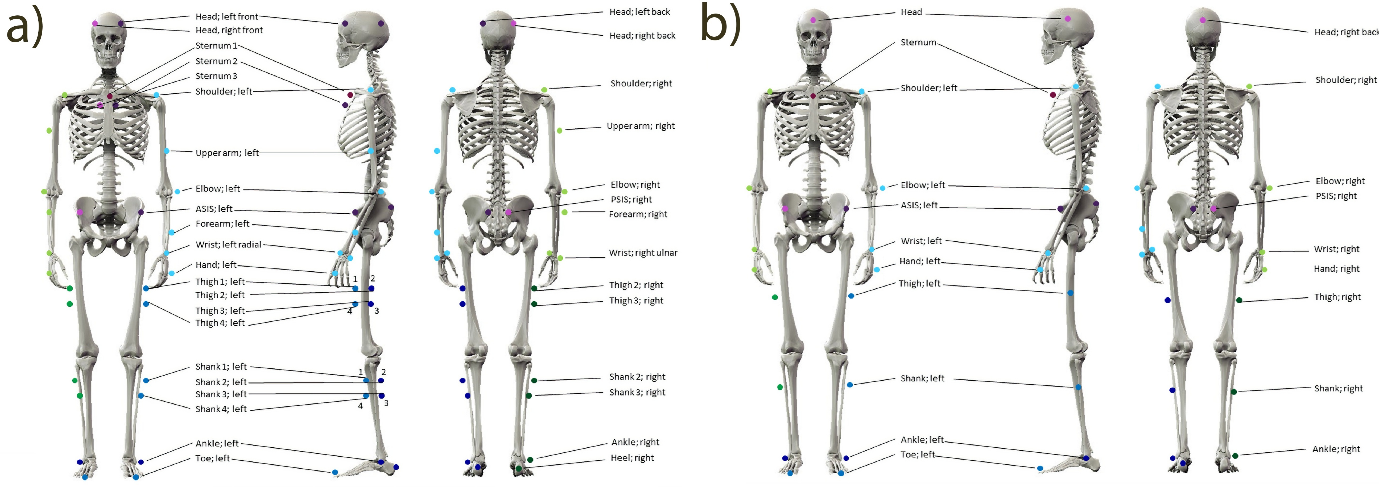


Supplementary Figure 2. Nodal strength of the body segments in Community 2 in AP direction. Asis – anterior superior iliac spine; fast – fast walking speed; las – least affected side, mas – most affected side, pref – preferred walking speed, psis – posterior superior iliac spine.

Supplementary Figure 1. a) Original marker positions; b) Marker positions after reduction. Figures adapted from^20^ with permission.


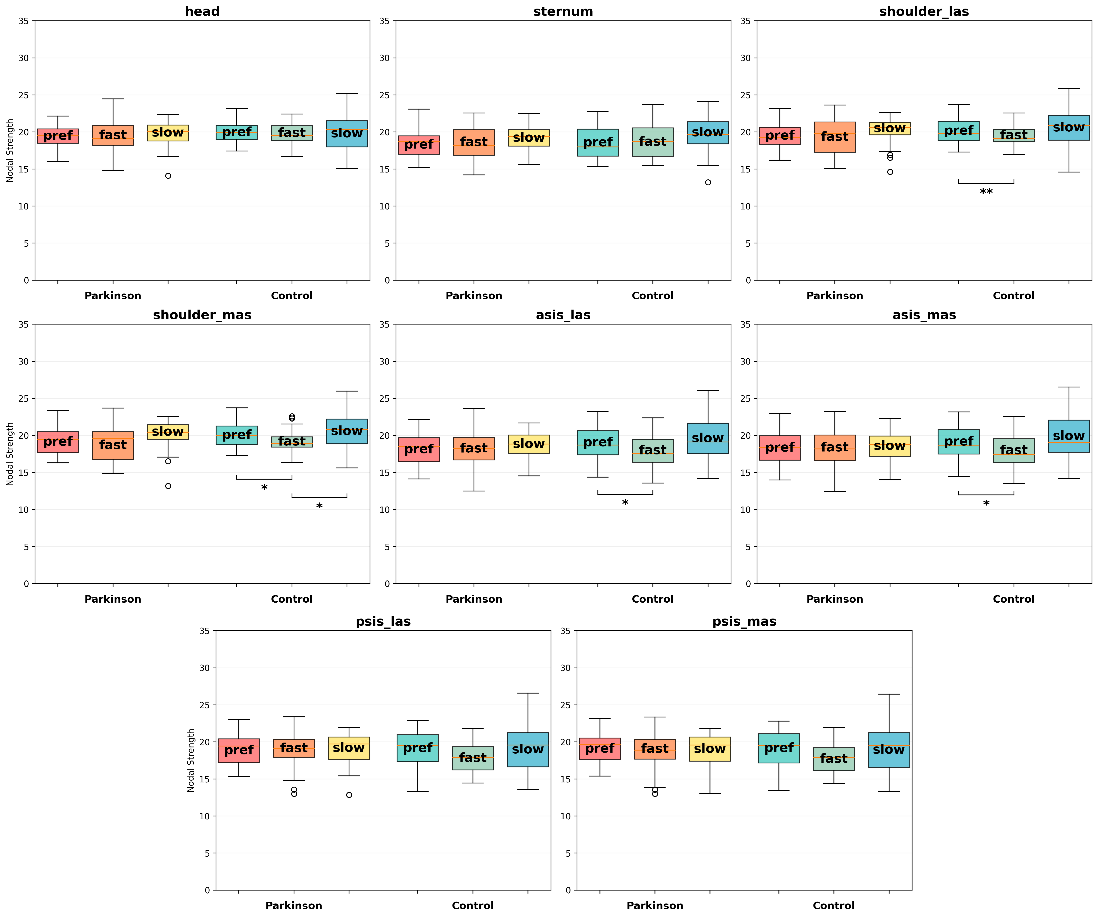

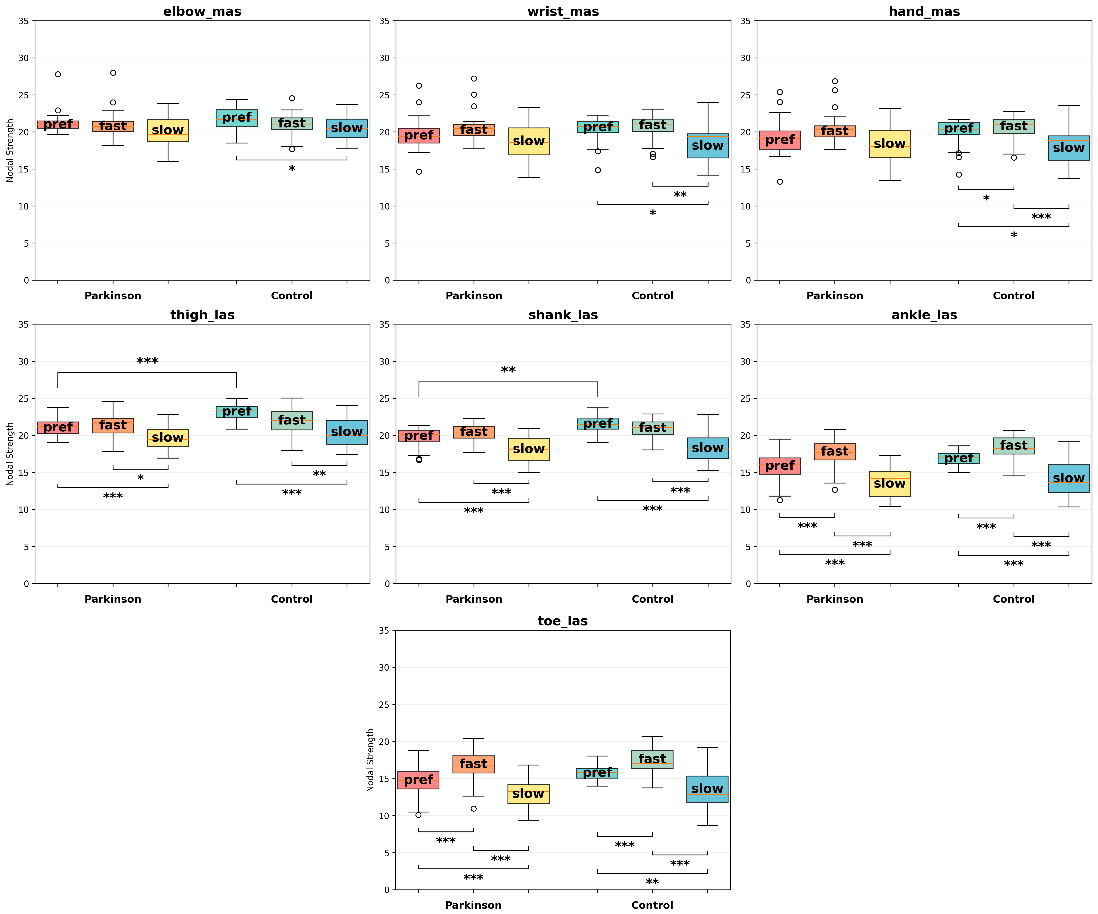


Supplementary Figure 4. Nodal strength of the body segments in Community 1 in ML direction. Asis – anterior superior iliac spine; fast – fast walking speed; las – least affected side, mas – most affected side, pref – preferred walking speed, psis – posterior superior iliac spine.

Supplementary Figure 3. Nodal strength of the body segments in Community 3 in AP direction. Asis – anterior superior iliac spine; fast – fast walking speed; las – least affected side, mas – most affected side, pref – preferred walking speed, psis – posterior superior iliac spine.


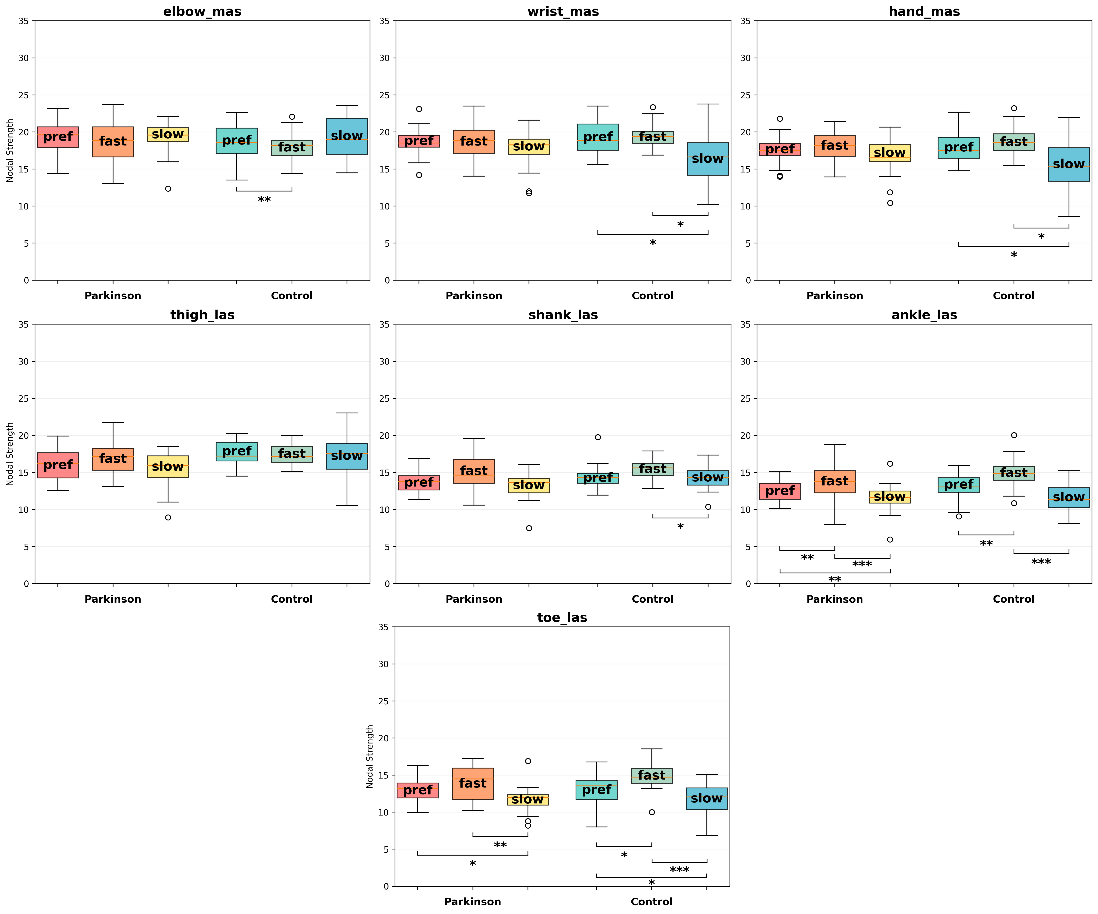

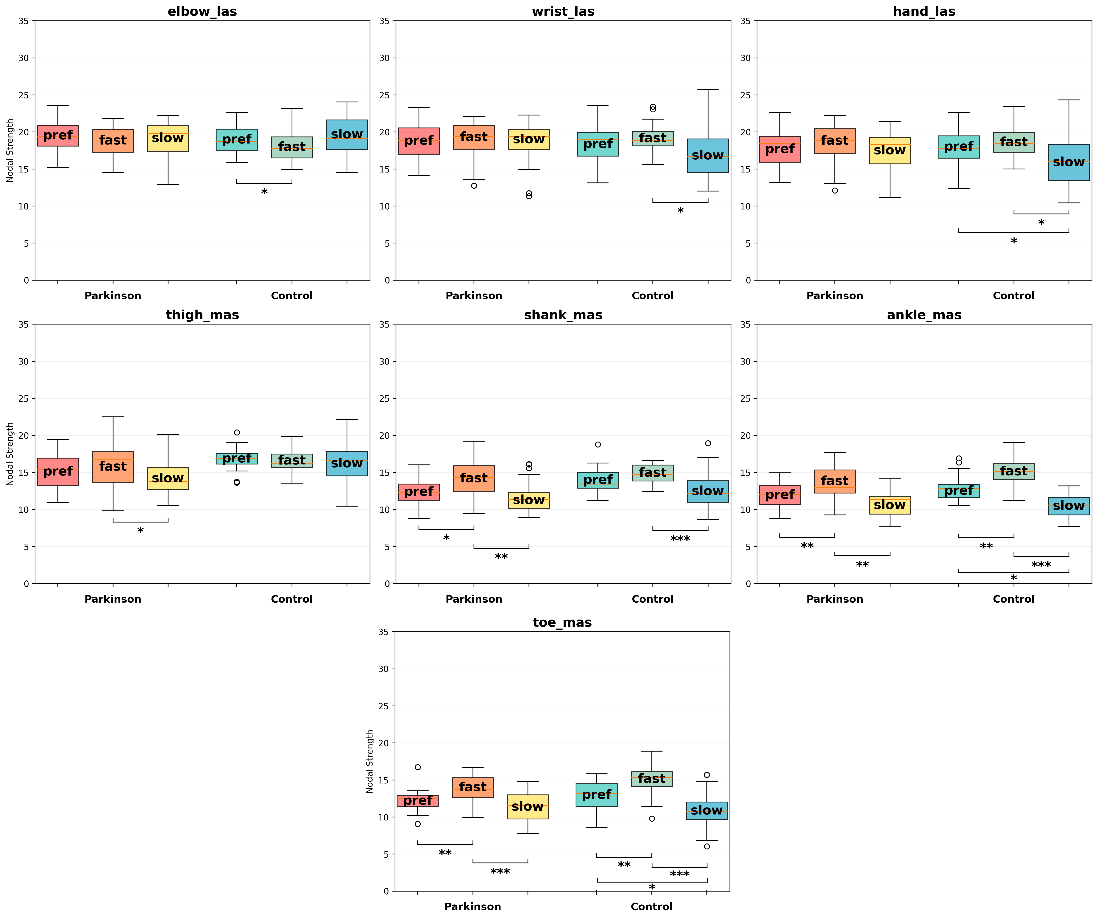


Supplementary Figure 6. Nodal strength of the body segments in Community 3 in ML direction. Asis – anterior superior iliac spine; fast – fast walking speed; las – least affected side, mas – most affected side, pref – preferred walking speed, psis – posterior superior iliac spine.

Supplementary Figure 5. Nodal strength of the body segments in Community 2 in ML direction. Asis – anterior superior iliac spine; fast – fast walking speed; las – least affected side, mas – most affected side, pref – preferred walking speed, psis – posterior superior iliac spine.


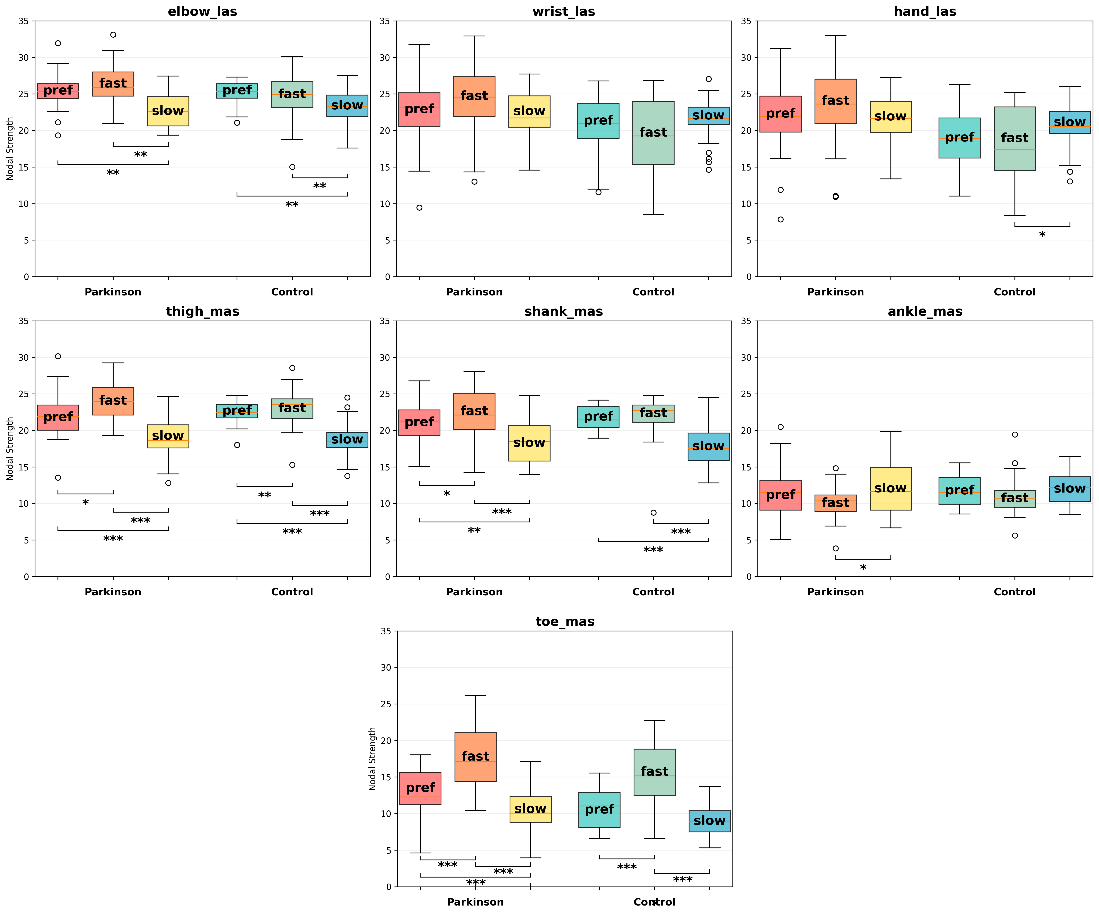

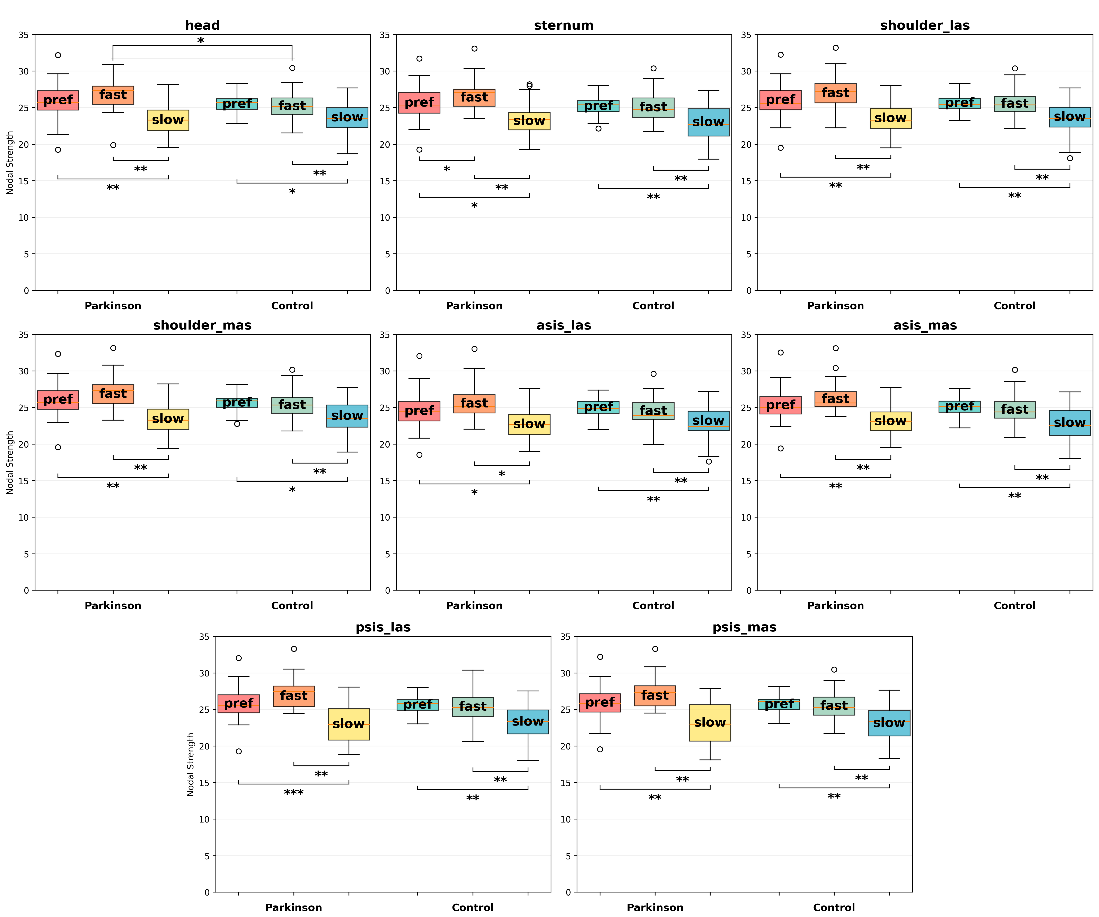


Supplementary Figure 8. Nodal strength of the body segments in Community 2 in V direction. Asis – anterior superior iliac spine; fast – fast walking speed; las – least affected side, mas – most affected side, pref – preferred walking speed, psis – posterior superior iliac spine.

Supplementary Figure 7. Nodal strength of the body segments in Community 1 in V direction. Asis – anterior superior iliac spine; fast – fast walking speed; las – least affected side, mas – most affected side, pref – preferred walking speed, psis – posterior superior iliac spine.


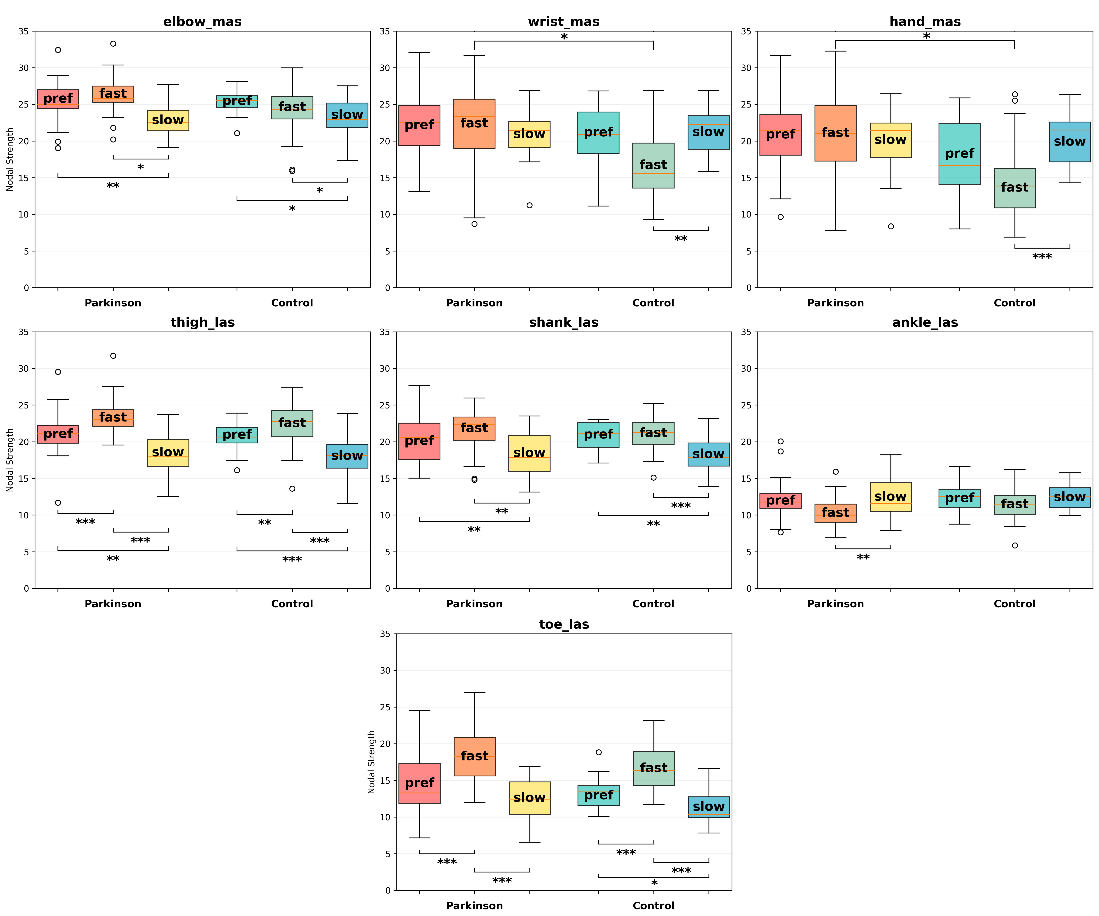


Supplementary Figure 9. Nodal strength of the body segments in Community 3 in V direction. Asis – anterior superior iliac spine; fast – fast walking speed; las – least affected side, mas – most affected side, pref – preferred walking speed, psis – posterior superior iliac spine.
